# 2例免疫检查点抑制剂联合化疗引发2型糖尿病个案报道

**DOI:** 10.3779/j.issn.1009-3419.2022.102.10

**Published:** 2022-04-20

**Authors:** 平 肖, 琳琳 张, 瑜 王, 凡路 孟, 鑫 王, 殿胜 钟

**Affiliations:** 1 300052 天津，天津医科大学总医院肿瘤内科 Department of Medical Oncology, Tianjin Medical University General Hospital, Tianjin 300052, China; 2 300052 天津，天津医科大学总医院内分泌代谢科 Department of Medical Endocrine Metabolism, Tianjin Medical University General Hospital, Tianjin 300052, China

**Keywords:** 免疫检查点抑制剂, 不良反应, 2型糖尿病, Immune checkpoint inhibitors, Adverse reaction, Type 2 diabetes mellitus

## Abstract

免疫检查点抑制剂（immune checkpoint inhibitors, ICIs）已经成为肿瘤治疗的重要手段，其在临床中的应用越来越广泛。ICIs引起的不良反应逐渐被认识，其中免疫治疗相关性糖尿病是罕见的不良反应，最常见的是1型糖尿病。随着ICIs联合化疗在肺癌患者中广泛应用，治疗过程中出现2型糖尿病的患者逐渐被发现，但是这部分患者后续继续应用ICIs维持治疗对血糖、ICIs治疗过程的影响尚不清楚。本文报道2例ICIs联合化疗期间新发的2型糖尿病，其中1例患者转为1型糖尿病，旨在增加对免疫治疗相关性糖尿病的认识。

## Abstract (en)

Immune checkpoint inhibitors (ICIs) have become an important means of cancer treatment, and their application in the clinic is becoming more and more widespread. The adverse reactions caused by ICIs are gradually recognized. Among them, immunotherapy-related diabetes is a rare adverse reaction and type 1 diabetes mellitusis common. With the wide application of ICIs combined with chemotherapy in lung cancer patients, patients with type 2 diabetes mellitus have gradually been discovered during the treatment. However, the effect of continued use of ICIs maintenance therapy on blood glucose and ICIs treatment process in these patients is still unclear. This article reports two cases of type 2 diabetes mellitus induced by immune checkpoint inhibitor combined with chemotherapy, one of whom converted to type 1 diabetes mellitus, in order to increase the understanding of immunotherapy-related diabetes.

免疫检查点抑制剂（immune checkpoint inhibitors, ICIs）已经成为肿瘤治疗的热点，主要包括：程序性死亡蛋白-1（programmed cell death protein-1, PD-1）抗体、程序性死亡配体1（programmed cell death ligand-1, PD-L1）抗体以及细胞毒性T淋巴细胞相关抗原4（cytotoxic T lymphocyte associated antigen 4, CTLA 4）抗体。随着ICIs在临床的广泛应用，这些药物带来的不良反应逐渐被认识。ICIs相关不良反应的发生主要与全身多脏器的免疫稳态紊乱有关，常见受累脏器包括皮肤、胃肠道、内分泌系统、肺、骨骼肌肉等，少见受累的脏器包括心血管、神经、血液等^[1]^。免疫治疗相关性糖尿病是一种罕见的不良反应，发生率低，在临床工作中往往得不到重视。目前ICIs联合化疗在肺癌一线治疗的应用越来越广泛，治疗期间部分患者会出现新发2型糖尿病（type 2 diabetes mellitus, T2DM）。但是这部分患者后续继续应用ICIs对血糖的影响尚不清楚。本文报告2例ICIs联合化疗治疗期间新发T2DM的诊治过程，旨在增加对免疫治疗相关性糖尿病的认识。

## 1 病例资料

病例1，男，55岁，2021年3月出现腰痛，查腰椎磁共振成像（magnetic resonance imaging, MRI）示胸、腰椎多发骨质破坏伴肿块影。胸部计算机断层扫描（computed tomography, CT）示右肺下叶软组织结节，并右肺门、纵隔淋巴结肿大。头MRI、腹部CT未见转移。气管镜检查提示：右中间干狭窄（于该处取活检）。病理回报：小细胞癌。既往史体健，否认高血压、糖尿病、冠心病等病史。入院诊断：广泛期小细胞肺癌。2021年3月19日开始给予一线阿替利珠单抗+依托泊苷+卡铂化疗。2个周期评效达到部分缓解（partial response, PR），4个周期后颅外维持PR，颅内考虑转移。2021年6月29日行全脑放疗。同时继续予阿替利珠单抗维持治疗。治疗期间出现免疫治疗相关性甲减，予左甲状腺素钠片替代治疗，目前病情评估维持PR。

糖尿病情况：2021年4月12日患者第2次阿替利珠单抗+化疗前，查空腹血糖8.3 mmol/L，三餐后血糖分别为9.9 mmol/L、10.2 mmol/L、8.9 mmol/L，尿酮体（-），糖化血红蛋白7.1%，空腹C肽1.79 ng/mL（0.78 ng/mL-5.19 ng/mL），口服糖耐量试验（oral glucose tolerance test, OGTT）检查提示糖尿病，胰岛素高峰延迟（表 1）。患者无糖尿病病史，否认糖尿病家族病史，体质指数（body mass index, BMI）18.5 kg/m^2^，化疗前血糖正常，治疗期间无口干、多饮、多尿、消瘦，无恶心呕吐，无发热、感染等应激状况，胰岛细胞抗体、谷氨酸脱羧酶抗体结果均为阴性。考虑患者为T2DM，予伏格列波糖口服降糖治疗，并继续给予阿替利珠单抗+化疗。2021年9月患者阿替利珠单抗维持治疗期间出现血糖控制不佳，空腹血糖8 mmol/L-11 mmol/L，餐后2 h血糖16 mmol/L-20 mmol/L，无酮症酸中毒等情况出现，遂联合德谷胰岛素、门冬胰岛素降糖治疗。复查OGTT提示C肽水平降低，考虑内源性胰岛素分泌不足（表 1），同时完善锌转运体-8抗体、酪氨酸磷酸酶抗体IA-2Ab、胰岛细胞抗体、谷氨酸脱羧酶抗体、HLA-DR4-DNA，结果均为阴性。目前患者仍在继续阿替利珠单抗维持治疗中，血糖经上述降糖方案治疗后未出现糖尿病急性并发症（图 1）。

**表 1:** 病例1 OGTT结果 The results of the 75 g OGTT of patient 1

	The initial diagnosis of diabetes						The result during the follow-up					Ref
	0 min	30 min	60 min	120 min	180 min		0 min	30 min	60 min	120 min	180 min	
Plasma glucose (mmol/L)	6.26	10.99	13.21	11.18	8.07		7.97	15.82	18.01	20.86	19.7	3.6-5.8
Serum immunoreactive insulin (mU/L)	3.7	26.8	45.60	49.6	26.8		108.0	113.6	96.3	112.2	83.4	4.0-18.0
C-peptide immunoreactivity (ng/mL)							0.58	1.46	1.63	4.44	2.44	0.78-5.19
OGTT: oral glucose tolerance test.												

**图 1:**
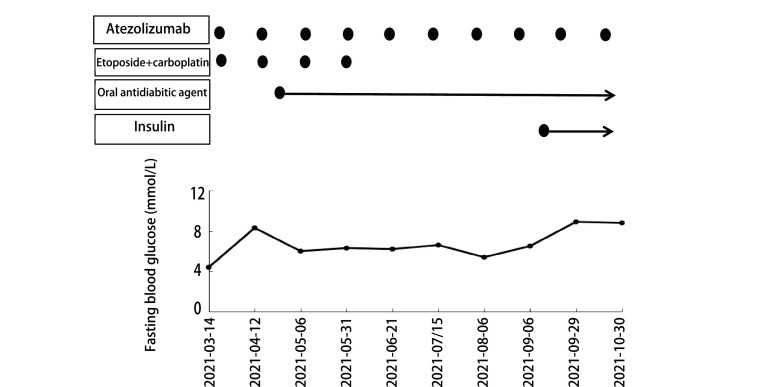
病例1治疗过程中空腹血糖变化情况 Changes of fasting blood glucose during treatment of patient 1

病例2，男，59岁，2020年4月发现右侧胸壁肿块，查胸部CT示右肺上叶可见57 mm×43 mm不均质软组织密度肿物，纵隔淋巴结肿大，右侧前胸壁可见不均质肿物影，考虑右肺上叶恶性肿瘤性病变伴右前壁、纵隔淋巴结转移。腹部CT示左侧肾上腺分歧部类圆形低密度结节影，考虑转移性病变。头MRI、骨发射型计算机断层扫描仪（emission computed tomography, ECT）未见转移。右侧胸壁肿物穿刺活检病理：肉瘤样癌。基因检测无热点基因突变及扩增，PD-L1肿瘤比例评分（tumor proportion score, TPS）：85%（使用22C3抗体）。既往“乙型肝炎”病史40余年，高血压病史4余年，否认糖尿病病史。入院诊断：Ⅳ癌。2020年6月-2020年10月，给予信迪利单抗+力朴素+卡铂化疗6个周期，2个周期后达到PR，6个周期后维持PR。2020年11月给予信迪利单抗200 mg d1 *Q3W*维持治疗。

糖尿病情况：2020年9月15日患者行第5周期信迪利单抗+化疗后，查空腹血糖6.8 mmol/L，未予重视。2020年11月29日空腹血糖12.2 mmol/L，三餐后血糖分别为15.2 mmol/L、16.2 mmol/L、15.3 mmol/L，尿糖3+，尿酮体（-），糖化血红蛋白7.3%，OGTT提示高胰岛素血症，C肽在正常范围（表 2）。患者无糖尿病病史及家族史，BMI 22 kg/m^2^，治疗前血糖正常，治疗期间无口干、多饮、多尿、消瘦，无恶心呕吐，无发热、感染等应激状况。完善锌转运体-8抗体、酪氨酸磷酸酶抗体IA-2Ab、胰岛细胞抗体、谷氨酸脱羧酶抗体、人类白细胞抗原（human leukocyte antigen, HLA）-DR4-DNA，结果均为阴性。考虑患者为T2DM，予西格列汀、恩格列净口服降糖治疗，治疗上继续给予信迪利单抗，每3个月左右复查1次胰岛素抗体。截止2021年6月，患者已经完成15个周期信迪利单抗的治疗，在上述口服降糖药物治疗期间，血糖控制平稳，未出现糖尿病酮症及酸中毒，且C肽一直在正常范围（图 2），胰岛素抗体均为阴性。

**表 2:** 治疗过程中病例2 OGTT结果 The results of the 75 g OGTT of patient 2 during the treatment

	0 min	30 min	60 min	120 min	180 min	Ref
Plasma glucose (mmol/L)	6.13	10.65	15.34	14.78	10.85	3.6-5.8
Serum immunoreactive insulin (mU/L)	11.0	47.4	130.4	207.1	83.6	4.0-18.0
C-peptide immunoreactivity (ng/mL)	2.59	5.91	11.28	16.14	12.33	0.78-5.19

**图 2:**
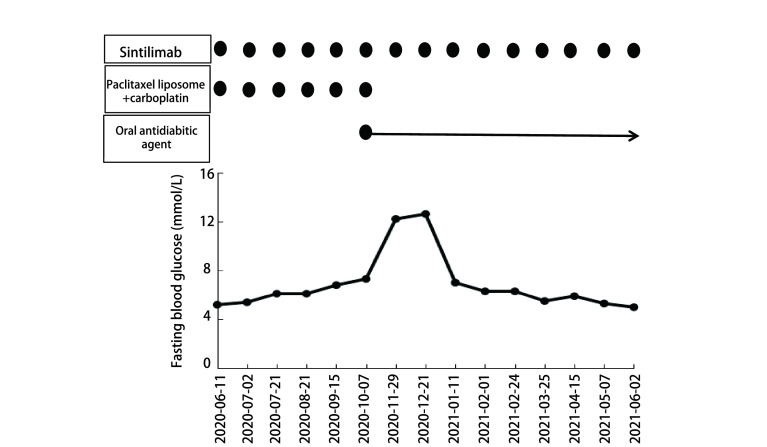
病例2治疗过程中空腹血糖变化情况 Changes of fasting blood glucose during treatment of patient 2

## 2 讨论

ICIs相关性糖尿病是ICIs治疗过程中罕见的不良反应。既往文献报道其发病率在0.2%-2%^[3, 4]^，且半数以上以糖尿病酮症起病^[2, 5]^。ICIs引起糖尿病多为T1DM，T2DM的报道更为罕见^[6]^。2018年一项荟萃分析^[6]^发现132例患者应用ICIs治疗过程中，出现1例T2DM。该患者在应用ICIs 24个月后出现T2DM，口服二甲双胍控制血糖，并继续接受了15个月ICIs的治疗。

ICIs引起的T1DM特点主要包括：①起病迅速，血糖波动大，短时间内出现糖尿病酮症酸中毒，易危及生命^[7]^；②胰岛功能衰竭快：当出现ICI-T1DM时，C-肽水平极低或检测不到^[8]^；③胰岛自身抗体阳性：半数ICI-T1DM患者检测到胰岛自身抗阳性^[9]^；④治疗依赖胰岛素替代治疗，并且≥2级考虑暂停ICIs直到血糖控制恢复到1级。而T2DM为胰岛素抵抗为主伴胰岛素进行性分泌不足，起病隐匿，症状轻或无症状，很少发生糖尿病酮症酸中毒及糖尿病昏迷等急性并发症。对于症状不显著的患者，口服降糖药为初始选择。当较大剂量口服多种降糖药物后血糖仍不达标时可考虑应用胰岛素替代治疗。

ICIs与T2DM的发生机制目前尚不清楚。研究^[10, 11]^表明，ICIs相关性糖尿病为一种炎症性疾病，由ICIs介导的多种因素对胰岛部分的破坏造成。T2DM被认为是一种代谢性疾病，而免疫稳态失衡在T2DM发病过程中起着重要作用。免疫应答过程激活后，调控炎性细胞信号转导，激活下游炎症反应，促进胰岛素敏感组织的炎症，进而产生胰岛素抵抗。胰岛素抵抗持续诱发低度炎症，使巨噬细胞、T淋巴细胞、B淋巴细胞等浸润内脏组织，诱发胰岛的炎症，最终引起胰岛β细胞的功能缺陷，出现T2DM^[12, 13]^。因此，ICIs可能通过激活体内的细胞免疫，造成胰岛素敏感组织的炎症反应，诱发胰岛素抵抗，形成T2DM，但是具体机制有待进一步研究证实。

在免疫联合化疗治疗过程中，造成患者血糖升高可能为1种因素或多种因素共同引起的。化疗药物也可以引起糖尿病的发生，尤其是顺铂或紫杉类药物，其中部分患者在停用化疗药物后血糖可恢复正常。此外，长期服用糖皮质激素同样可以引起糖尿病的发生，但是糖耐量正常的患者发展为糖尿病并不常见^[14]^。糖皮质激素引起的糖尿病以午餐后至睡前血糖升高为主要表现，空腹血糖一般正常，并且高血糖多在停药后48 h明显减弱或消失。2例患者化疗过程中曾短期口服地塞米松片（6 mg d1, 3.75 mg d2-d4）止吐治疗，未长期服用糖皮质激素，并且以空腹血糖升高为主。因此，糖皮质激素引起糖尿病可能性小。不过2例患者均使用ICI联合铂类化疗，其中1例同时使用紫杉类药物，因此，造成患者T2DM的出现可能是多种因素共同作用所致。

研究^[2]^发现既往合并T2DM的患者在接受ICIs治疗后部分患者可能出现胰岛功能衰竭，发展为ICI-T1DM。然而ICIs联合化疗治疗过程中新出现的T2DM，目前的文献报告尚未观察到转为T1DM。病例1初始胰岛功能正常，在随访的过程中出现血糖控制不佳，OGTT提示C肽水平降低，内源性胰岛功能障碍，考虑为ICIs造成的自身免疫性胰腺的β细胞功能损伤，后续发展为ICI-T1DM。病例2在出现T2DM后随访的8个月内尚未出现胰岛衰竭的表现。因此，在随访的过程中，我们除了需要监测患者的临床症状、血糖、尿酮体外，还要关注胰岛功能（OGTT、C肽）、胰岛相关抗体等情况，一旦发展为T1DM，及时采取措施，避免急性并发症的发生。目前临床实践中，使用ICIs前并未常规监测OGTT及糖化血红蛋白等，当发现空腹血糖升高时，进而才完善相关糖尿病检查，这可能会造成少部分患者在治疗过程中具体的血糖升高时间不容易判断的情况。

基于文献回顾和本病例，提醒我们，在ICI联合化疗治疗过程中，有可能会出现隐匿的T2DM。与ICI-T1DM相比，T2DM起病缓，口服降糖药物控制血糖良好，较少引起急性并发症，不会影响ICIs的治疗过程。对于这部分患者我们在随访过程中，要警惕其是否可能会转化为T1DM。
